# Biological and structural properties of curcumin-loaded graphene oxide incorporated collagen as composite scaffold for bone regeneration

**DOI:** 10.3389/fbioe.2024.1505102

**Published:** 2024-11-20

**Authors:** Qi Xie, Tianqi Wang, Lina He, Hongbo Liang, Jingxuan Sun, Xiaoxiao Huang, Weili Xie, Yumei Niu

**Affiliations:** ^1^ The First Affiliated Hospital of Harbin Medical University, School of Stomatology, Harbin Medical University, Harbin, China; ^2^ School of Materials Science and Engineering, Harbin Institute of Technology, Harbin, China

**Keywords:** collagen, graphene oxide, curcumin, bone regeneration, antibacterial

## Abstract

**Introduction:**

To address the challenges related to bone defects, including osteoinductivity deficiency and post-implantation infection risk, this study developed the collagen composite scaffolds (CUR-GO-COL) with multifunctionality by integrating the curcumin-loaded graphene oxide with collagen through a freeze-drying-cross-linking process.

**Methods:**

The morphological and structural characteristics of the composite scaffolds were analyzed, along with their physicochemical properties, including water absorption capacity, water retention rate, porosity, *in vitro* degradation, and curcumin release. To evaluate the biocompatibility, cell viability, proliferation, and adhesion capabilities of the composite scaffolds, as well as their osteogenic and antimicrobial properties, in vitro cell and bacterial assays were conducted. These assays were designed to assess the impact of the composite scaffolds on cell behavior and bacterial growth, thereby providing insights into their potential for promoting osteogenesis and inhibiting infection.

**Results:**

The CUR-GO-COL composite scaffold with a CUR-GO concentration of 0.05% (w/v) exhibits optimal biological compatibility and stable and slow curcumin release rate. Furthermore, *in vitro* cell and bacterial tests demonstrated that the prepared CUR-GO-COL composite scaffolds enhance cell viability, proliferation and adhesion, and offer superior osteogenic and antimicrobial properties compared with the CUR-GO composite scaffold, confirming the osteogenesis promotion and antimicrobial effects.

**Discussion:**

The introduction of CUR-GO into collagen scaffold creates a bone-friendly microenvironment, and offers a theoretical foundation for the design, investigation and utilization of multifunctional bone tissue biomaterials.

## 1 Introduction

Bone defects occurring in the oral and maxillofacial region, often induced by trauma, tumors and inflammation, not only create aesthetic problems in the face, but also influence articulation and mastication. While minor bone defects might self-heal, the healing process is time-consuming with a risk of infection. For large bone defects, the treatment usually involves autologous or allogeneic bone grafting. However, autologous bone grafting requires an additional surgery to obtain grafts, leading to potential complications such as inflammation and infection. It is further limited by donor availability. Conversely, the clinical application of allogeneic bone grafting is restricted due to the insufficient osteogenic capacity and varying levels of immune rejection of allogeneic bone (Bose and Sarkar, 2020).

In recent years, the application of biomimetic bone materials in the field of bone defect treatment has become increasingly widespread due to their superior biological activity and physicochemical properties, offering clinicians more options and significantly enhancing treatment outcomes (Rifai et al., 2023; Yang et al., 2023). Collagen (COL), a central component of skin, bones, tendons, and ligaments, has emerged as a favored natural biomaterial in bone tissue engineering due to its excellent biocompatibility, high porosity, ease of integration with other materials, hydrophilicity, and low antigenicity (Gu et al., 2019). Recent research indicates that the rapid preparation of porous collagen membranes can be achieved through the combination of bioblasting and ultrasonic treatment techniques. This method not only streamlines the preparation process but also endows collagen membranes with a porous structure, facilitating cell attachment and proliferation (Wu et al., 2021). However, collagen, as an independent material, is insufficient to meet the current demand for the repair of bone tissue defects, hence prompting the development of various collagen-containing composite scaffolds (Quinlan et al., 2015; Zeng et al., 2020).

Graphene oxide (GO), known for its excellent mechanical properties, has been combined with numerous biomaterials to enhance their structure integrity (Dinescu et al., 2014; Luo et al., 2015; Najafinezhad et al., 2022; Yu et al., 2023). In addition, the abundance of oxygen-containing functional groups on the surface of graphene oxide not only enhances the hydrophilicity, but also promotes binding with biomolecules and polymers for use in combination with other biomaterials and drugs (Pan et al., 2012). Moreover, the large specific surface area of graphene oxide allows for the absorption of proteins and adherent cells, promoting osteogenic differentiation of stem cells, and establishing it as a promising candidate for bone tissue regeneration (Choe et al., 2019; Zheng C. X. et al., 2020).

The emergence of scaffold materials not only facilitates bone reconstruction but also provides a robust platform for targeted delivery of therapeutic agents, enabling sustained and gradual release of drugs within defect areas, thereby expediting bone healing (Astaneh et al., 2024). However, the challenge of identifying safer, lower-side-effect drugs that effectively repair bone defects remains a focal point for researchers. Curcumin, a natural polyphenol renowned for its anti-inflammatory, antibacterial, and antioxidant properties, exhibits significant potential in regulating bone cell proliferation, differentiation, and promoting bone formation (Li et al., 2021; Shakeri and Boskabady, 2017; Sun et al., 2022). Nonetheless, its application has been constrained by poor water solubility and low bioavailability. In recent years, scholars have dedicated efforts to improving curcumin’s solubility, stability, and bioavailability. Specifically, (Mitra et al., 2015) developed a 3D scaffold biomaterial tailored for wound healing by loading curcumin into fish scale collagen reinforced with nano-graphene oxide (GO). Additionally, other researchers designed cylindrical bone graft substitutes employing reduced graphene oxide (rGO) and nano-curcumin, which exhibit excellent physicochemical properties, biocompatibility, and antibacterial performance (Senthil et al., 2022). These advancements underscore the innovative potential of curcumin in bone tissue engineering. Therefore, the technique for effective loading of curcumin is focused and developed.

**FIGURE 1:**
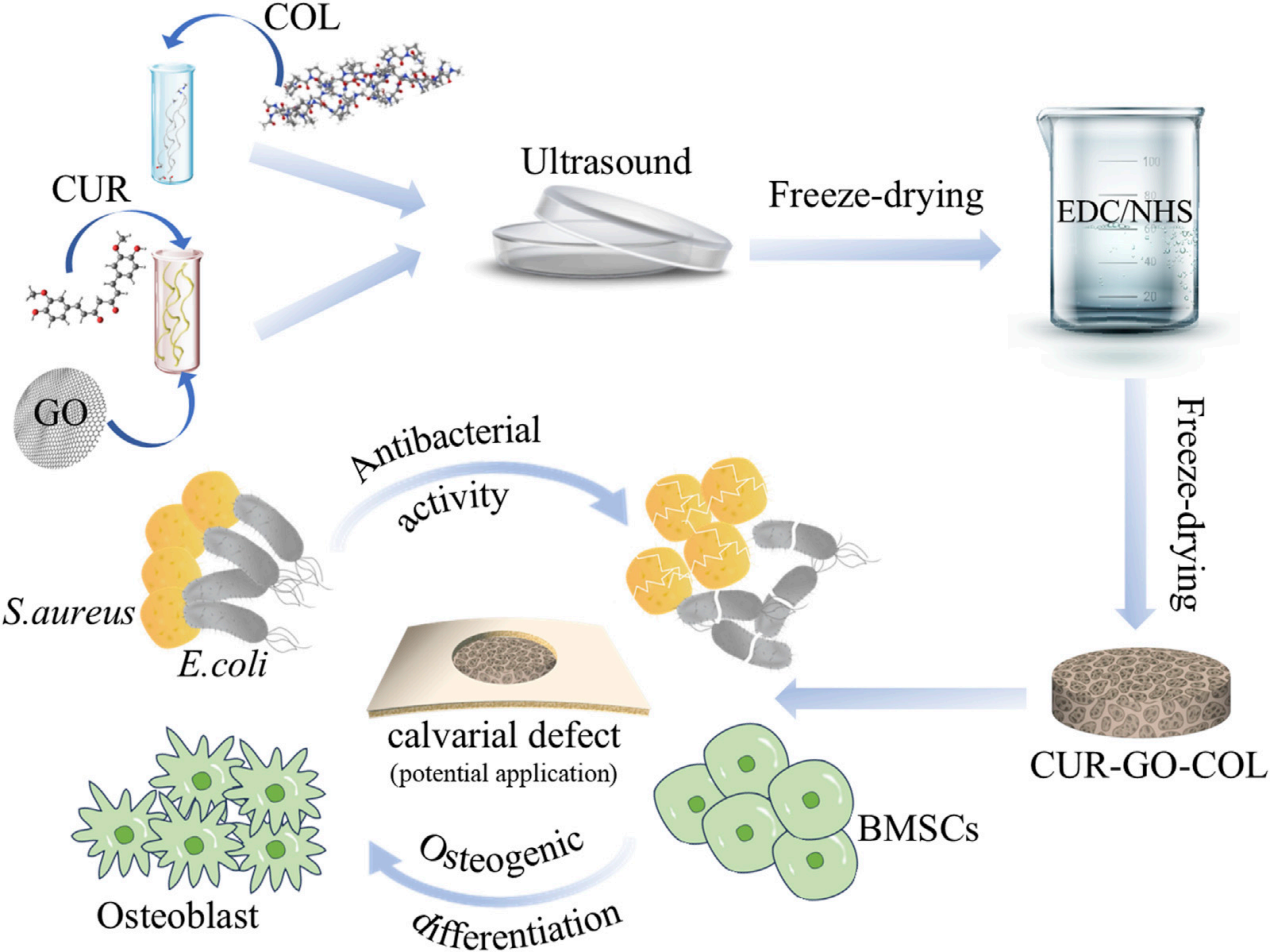
Schematic illustration of the fabrication of composite scaffold and their application *in vitro*.

In response to these challenges, a porous biocomposite based on CUR-GO-COL system for bone defect regeneration was developed. First, curcumin was loaded on GO, followed by the formation of a CUR-GO-COL complex scaffold with COL, which is rich in amino functional groups. This study not only evaluated the biocompatibility of the CUR-GO-COL composite scaffold, but also explored the synergistic effects in improving osteogenic properties and antibacterial capability of curcumin and GO in the composite scaffold, thus underscoring the potential of the composite in bone regeneration strategies (Figure 1).

## 2 Materials and methods

### 2.1 Materials and reagents

Graphite flakes, potassium permanganate, sulfuric acid (98.9%), phosphoric acid (99%), hydrogen peroxide (99.9%), hydrogen chloride (99%), acetic acid (99.9%) and other solvents were purchased from Sinopharm Chemical Reagent (China); Type I collagen, curcumin (94%), N-(3- Dimethylaminopropyl)-N-ethylcarbodiimide hydrochloride crystalline (EDC) and N-hydroxysuccinimide (NHS) were purchased from Sigma-Aldrich (United States); BMSCs cells purchased from Xiamen Yimo Biotechnology Co., Ltd. (China); Dulbecco’s Modified Eagle Medium/Nutrient Mixture F-12 (F12/DMEM) were purchased from Wuhan Pricella Biotechnology Co., Ltd. (China); Fetal bovine serum (FBS), penicillin-streptomycin (PS) and other reagents about cell culture were obtained from Gibco (United States).

### 2.2 Preparation of CUR-GO

In this experiment, graphene oxide (GO) was synthesized from high-purity graphite using the Hammers method (Hummers and Offeman, 1958). For loading of curcumin (CUR) on GO, firstly, a GO solution with a concentration of 2 mg/mL was stirred to ensure uniform dispersion. Secondly, various CUR solutions with concentrations ranging from 0.05 mg/mL to 1.0 mg/mL were prepared and mixed with the 2 mg/mL GO solution. This mixture was then placed in a shaker at 120 rpm overnight. Finally, the obtained solution was centrifuged at 10,000 rpm for 10 min to separate the loaded GO from the unbound CUR. The concentration of remaining curcumin in the supernatant was determined using UV–vis spectroscopy at a wavelength of 425 nm. Based on the plotted standard curve, the loading efficiency of CUR onto GO was calculated using the following Formula 1: where m_c_, m_s_ and m_GO_ are the masses of total curcumin, supernatant’s curcumin, and GO, respectively. (Supplementary Material)

$$\text{Loading rate of curcumin }\left(\%\right)=\frac{\text{mc}-\text{ms}}{\text{mGO}}\times 100$$ (1)

### 2.3 Preparation of CUR-GO-COL scaffold

In this experiment, the collagen (COL) scaffold was prepared using the conventional freeze-drying method. Initially, type I collagen was cut into pieces and dissolved in 0.05 M acetic acid to achieve at 1% w/v suspension, utilizing magnetic stirring for complete dissolution. This solution was then transferred into a mould and frozen at −20°C overnight. Following this, it was lyophilized by a freeze-drierat −50°C for 24 h. For cross-linking, the scaffold was immersed in a 95% v/v ethanol solution containing EDC and NHS (EDC: NHS ≥ 4), and cross-linked at ambient temperature for 24 h. Then, the sample was washed with deionized water several times, and lyophilized again for 24 h to obtain a porous COL scaffold.

To prepare a series of CUR-GO-COL composite scaffolds, the COL solution was mixed with varying concentrations of CUR-GO (0.025% w/v, 0.05% w/v, 0.075% w/v, 0.1% w/v, 0.125% w/v and 0.15% w/v), respectively. All mixture were ultrasonicated in an ice water bath for 60 min to ensure uniform dispersion. Next, these mixtures were subjected to the same cross-linking and wash steps as the COL scaffold. Finally, after a 24-h lyophilization procedure, a series of porous CUR-GO-COL scaffolds containing 0.025% w/v, 0.05% w/v, 0.075% w/v, 0.1% w/v, 0.125% w/v and 0.15% w/v CUR-GO were obtained and designated as 1CUR-GO-COL, 2CUR-GO-COL, 3CUR-GO-COL, 4CUR-GO-COL, 5CUR-GO-COL and 6CUR-GO-COL, respectively.

### 2.4 Materials characterization

#### 2.4.1 Morphology and structure

Based on the aforementioned material synthesis results, we selected four types of successfully fabricated scaffold materials (1CUR-GO-COL, 2CUR-GO-COL, 3CUR-GO-COL, and 4CUR-GO-COL) for subsequent testing and corresponding experimental investigations. The morphology of the graphene oxide (GO) and as-prepared scaffolds were observed by a scanning electron microscope (SEM; model-SUPRA55, ZEISS, Germany). Functional groups of samples were identified and analyzed via a Fourier Transform Infrared spectroscopy (FT-IR; IS 50, Thermo Fisher, United States). The spectra were collected from 4,000 to 500 cm^−1^with a resolution of 2 cm^−1^. The chemical composition of the samples was further analyzed through a Raman Spectra (RENISHAW, United Kingdom) using a 532 nm laser. The crystalline phases of as-preapred sample were identified using an X-ray diffractometer (XRD; X'PERT PRO, Nalytical, NL). The XRD patterns were recorded over a 2θ ranging from 5° to 50°.

#### 2.4.2 Porosity and water absorption measurements

The porosity of each scaffold was measured by the ethanol displacement method. Briefly, the radius (r), thickness (h) and weight (W_1_) of each dry scaffold were measured. Subsequently, the scaffolds were immersed in ethanol for 24 h, and the weight of the enthanol-saturated scaffold (W_2_) was measured. The porosity of as-prepared scaffolds was calculated using the following Formula 2:

$$\text{Porosity }\left(\%\right)=\left(W_{2}-W_{1}\right)/\left(\rho \times \pi \times r^{2}\right)\times h\times 100\%\;\left(\rho \text{ denotes ethanol density}\right)$$ (2)

The water absorption rate and retention rate of the scaffold were calculated according to the previous literature (Li et al., 2019). Briefly, each dry scaffold was weighted (W_1_), and then immersed in distilled water at room temperature for 24 h. After that, the sample was taken out, the surface water of the scaffold was absorbed by filter paper, and the wet weight (W_3_) of the sample was measured immediately. The scaffold was placed into a centrifuge tube and centrifuged at 500 rpm for 5 min, and then weighted again (W_4_). The following Formulas 3, 4 was used to calculate the water absorption rate and retention rate:

$$\text{Water absorption rate }\left(\%\right)=\left[\left(W_{3}-W_{1}\right)/\;W_{1}\right]\times 100\%$$ (3)

$$\text{Water retention rate }\left(\%\right)=\left[\left(W_{4}-W_{1}\right)/\;W_{1}\right]\times 100\%$$ (4)

### 2.5 Cytotoxicity assays

To further determine the appropriate concentration of CUR-GO, BMSCs were homogeneously inoculated at 96-well plates by 2,500 cells/well on col and col scaffolds with different concentrations of CUR-GO, and cultured for 1 and 4 days. After a specified period of time, the original culture solution was discarded, and the CCK-8 solution was mixed with the medium solution at a ratio of 1:10, 110 μL was added to each well, and cultivated for 2 h. The absorbance of each group of materials was detected at the wavelength of 450 nm using an enzyme labeling instrument.

### 2.6 *In vitro* degradation property

To investigate the *in vitro* degradation performance of composite materials, three groups of collagen scaffolds with an initial weight of W_1_were immersed in 10 mL of PBS. The collagen scaffolds were then placed in an environment maintained at 37°C. After 7, 14, 21, and 28 days, the scaffolds were removed, lyophilized, and weighed again to obtain W_5_. The PBS was replaced every 2 days. The degradation rate was calculated using the following Formula 5:

$$\text{Degradation rate }\left(\%\right)=\left(W5-W1\right)\;/W1\times 100\%$$ (5)

### 2.7 *In vitro* curcumin release property

The *in vitro* drug release test was conducted with the curcumin-loaded material by use of a constant temperature water bath shaker. The sample was immersed in 10 mL of PBS solution containing 30% alcohol. Next, 2 mL of the solution was taken at an appropriate time interval, and then 2 mL of PBS was added to the solution to keep a constant volume. The absorbance of the sample was measured by UV spectrophotometer at the wavelength of 425 nm, and the amount of released curcumin varying with the test time point was calculated depending on the previously plotted drug standard curve. Three parallel tests were conducted.

### 2.8 Biocompatibility assay of scaffolds

#### 2.8.1 Cell viability assay

BMSCs were inoculated onto three groups of different materials in 24-well plates at a density of 1 × 10^4^ cells/well, and cultured for 1 day, then the original medium was discarded, the cells were rinsed with sterile PBS for three times, and then 1 × Assay Buffer was mixed with Calcein-AM and Calcein-PI by the ratio of 1,000:1:1 in each well in accordance with the instructions of the live/dead cell staining kit, and then 250 μL of the mixture was added to each well. The wells were cultivated at 4°C for 30 min, protected from light, and the viability of the cells on the materials of each group was observed by fluorescence microscope.

#### 2.8.2 Cell adhesion assay

BMSCs were inoculated onto three groups of different materials in 24-well plates at a density of 1 × 10^4^ cells/well, and after 1 day of culture, the original culture medium was discarded, and the cells were rinsed with PBS for 3 times; then the cells were fixed using 4% paraformaldehyde for 15 min at room temperature, and the fixative was aspirated and the cells were rinsed with PBS for 2 times; and then the cells were reacted with 0.5% Triton X-100 for 10 min to increase the permeability, and the cells were rinsed twice with PBS; TRITC-Phalloidin working solution was added, and the cells were cultivated for 30 min at room temperature and protected from light. After the cells were rinsed twice with PBS, DAPI solution was added, and the cells were cultivated for 5 min at room temperature and protected from light. Finally, the cell adhesion behaviors were observed using a fluorescence inverted microscope.

#### 2.8.3 Cell proliferation assay

BMSCs were homogeneously inoculated into 96-well plates and three groups of different materials (COL, GO-COL, CUR-GO-COL) at 2,500 cells/well and cultivated for 1 and 4 days. After a specified period of time, the original culture solution was discarded, and the CCK8 solution was mixed with the medium solution at a ratio of 1:10 according to the CCK-8 instruction, 110 μL was added to each well, and cultivated for 2 h. The absorbance of each group of materials was detected at the wavelength of 450 nm using an enzyme labeling instrument. The cell viability was calculated according to the following Formula 6, A(substance): The absorbance value of the experimental sample; A(blank): The absorbance value of the blank control, which contains only the detection solution; A(control): The absorbance value of the control group without any material.

$$\text{Cell viability rate }\left(\%\right)=\left[A\left(\text{substance}\right)-A\left(\text{blank}\right)\right]/\;\left[A\left(\text{control}\right)-A\left(\text{blank}\right)\right]x\;100\%$$ (6)

### 2.9 *In vitro* cellular osteogenesis assay

#### 2.9.1 Alkaline phosphatase (ALP) activity

BMSCs were uniformly inoculated onto three different groups of materials in 24-well plates at a density of 1 × 10^4^/well, and after the wall-adherent cells had reached 70%–80% fusion, the mineralization-inducing solution was replaced for culture, followed by solution change every 2–3 days. After 7 days of osteogenic induction culture, staining and photographing were performed. Moreover, a quantitative analysis about the ALP activity was measured according to the specific experimental procedure of ALP Activity Assay Kit, and the absorbance value at 405 nm was measured by an enzyme marker. ALP activity was calculated from the linear equation plotted on the standard samples.

#### 2.9.2 Alizarin red (ARS) staining and semi-quantitative analysis

BMSCs were uniformly inoculated onto three different groups of materials in 4-well plates at a density of 1 × 10^4^ cells/well; and after the wall-adherent cells had reached 70%–80% fusion, the mineralization-inducing solution was replaced for culture, followed by a fluid change every 2–3 days. After 21 days of osteogenic induction culture, the original culture solution was discarded and the cells were washed twice with PBS, 4% paraformaldehyde was added, and then washed twice with PBS. 500 μL of alizarin red dye solution was added to each well, and then the cells were stained for 10 min at room temperature and protected from light. The alizarin red dye solution was discarded, and cells were washed gently with PBS for 2 times, and photographed and observed by a microscope. For semi-quantitative detection of extracellular matrix mineralization, 250 μL of prepared 10% cetylpyridinium chloride was added to each well; uniform shaking at room temperature was conducted; then 100 μL was pipetted from each well and added into a 96-well plate; and the absorbance value at 630 nm was measured by an enzyme marker.

### 2.10 *In vitro* antibacterial activity

In order to evaluate antibacterial abilities, *E. coli* (ATCC25922) and *Staphylococcus aureus* (ATCC25923) were taken out from a refrigerator at −80°C for streak recovery, single colonies were picked and cultivated and passaged at 37°C under oscillation at 130 r/min, and the bacterial suspension was diluted to 1 × 10^6^ CFU/mL for subsequent experiments.

#### 2.10.1 Growth curves of bacteria

The three groups of materials were immersed in a bacterial suspension of 1 × 10^6^ CFU/mL, placed in a constant temperature shaker, and cultivated with oscillation at 37°C and 130 r/min for a total of 12 h. The bacterial suspension without the addition of any material was used as the control group. At the set time, 150 µL of the bacterial suspension was pipetted into a 96-well plate, and the optical density (OD) value of the bacterial suspension at 600 nm was measured by using an enzyme marker to reflect the concentration of bacteria at this time.

#### 2.10.2 Plate antibacterial experiment

The three groups of materials were co-cultured with bacteria for 24 h; 60 µL of bacterial solution was extracted and diluted to a specific concentration; 100 µL of the diluted bacterial solution was transferred to the LB solid medium plate, evenly spread on the solid plate, and cultivated at 37°C for 12 h; and the culture plate was taken out and the colonies were photographed inside the plate.

### 2.11 Statistical analysis

All data from the repeated experiments are presented as the mean ± standard deviation and were analyzed using one-way ANOVA and Tukey’s HSD (GraphPad Prism 8, United States). A value of *P* < 0.05 was considered to be statistically significant. (*, *P* < 0.05; * *, *P* < 0.01; * * *, *P* < 0.001).

## 3 Results

### 3.1 Surface morphology and composition of CUR-GO-COL scaffold

The morphology of the GO prepared by Hummer’s method was examined using SEM, as shown in Supplementary Figure S1. The SEM images exhibited that the as-prepared GO possesses well-defined edges and extensive 2D planes. The curcumin solution was added to the GO dispersion in a slow dropping manner, allowing the anchoring of curcumin by the functional groups on GO. When curcumin was loaded on GO, the results are shown in the Supplementary Figures S2, S3, with the increase of curcumin concentration, the drug load of CUR-GO first continuously increased linearly, reached a peak and then slowly decreased, and was finally stabilized. When the curcumin concentration exceeded 0.7 mg/mL, the loading rate of CUR-GO decreased slightly to 31.54%. Therefore, we chose the 0.7 mg/mL group for subsequent experimental studies.

The XRD pattern of as-prepared GO revealed a distinct diffraction peak at 2θ of 10.2°, corresponding to the (002) crystal plane of GO. The XRD pattern of the CUR-GO exhibited additional diffraction peaks between 2θ of 5°–30°, which can be attributed to the presence of curcumin (Supplementary Figure S4). Besides, the IR spectrum of GO displayed several distinctive vibrational peaks at 3,490 cm^−1^ (O-H), 1731 cm^−1^ (C=O), and peaks at 1,370 cm^−1^ and 1,070 cm^−1^ (C-O), as indicated in Supplementary Figure S5. In addition to the characteristic peaks of graphene oxide, the IR spectrum of the CUR-GO composite highlighted the characteristic infrared absorption peaks at 3,508 cm^−1^, 1,627 cm^−1^ and 1,602 cm^−1^, which are attributed to O-H, C=O and benzene rings from curcumin, respectively (Supplementary Figure S5). The Raman spectrum of GO exhibited two distinct peaks at 1,348 cm^-1^ and 1,590 cm^−1^, which correspond to the D and G peaks of GO, respectively. For the CUR-GO composite, alongside these two GO peaks, additional Raman peaks at 1,000 cm^−1^ and 1750 cm^−1^ were detected, which are associated with the CUR phase (Supplementary Figure S6). These observations confirmed the successful incorporation of curcumin into the GO framework within the CUR-GO composite.

**FIGURE 2:**
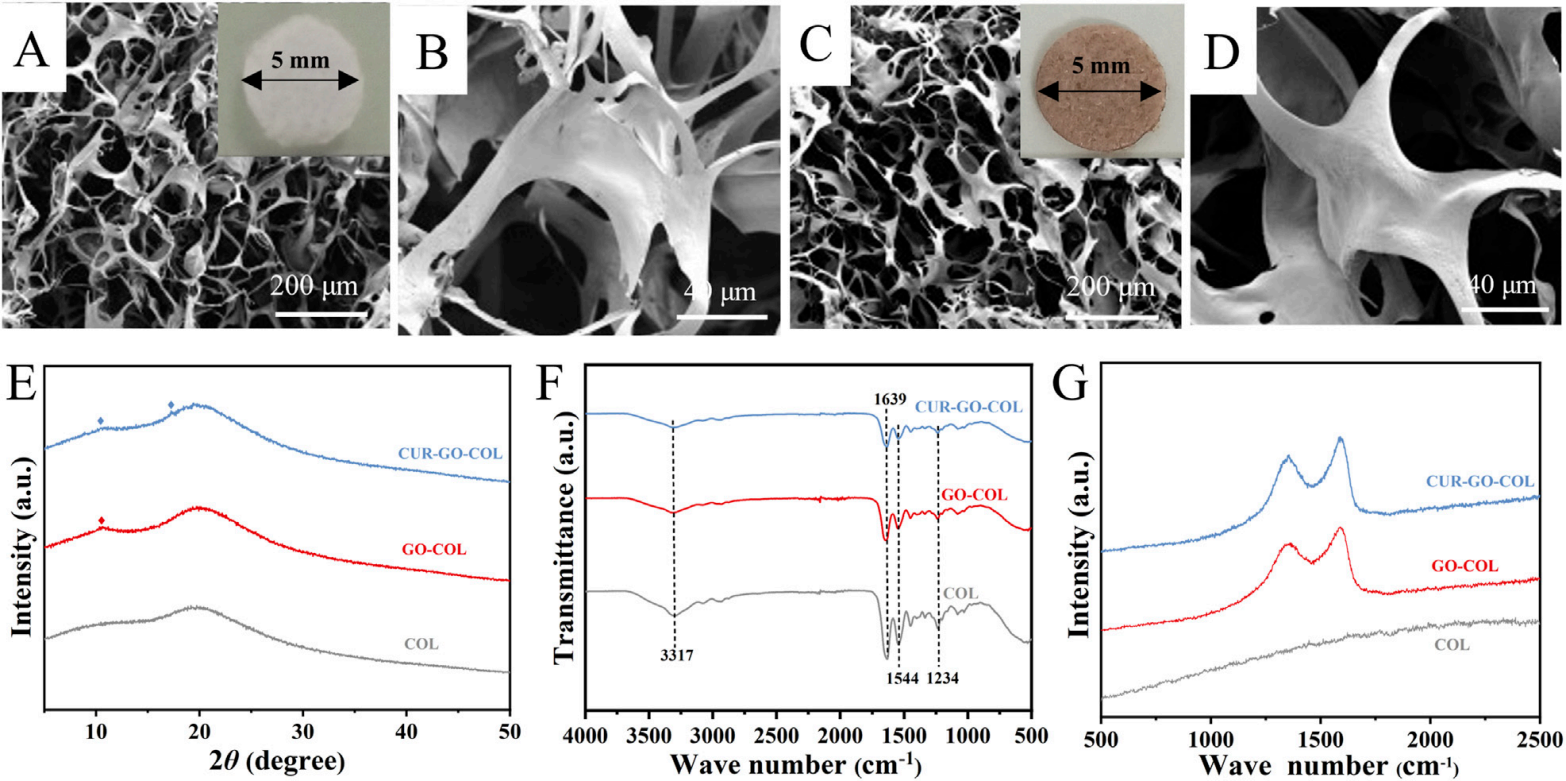
Morphology and structure of the CUR-GO-COL composite scaffold. Macroscopic view and SEM images of the COL scaffold **(A, B)** and CUR-GO-COL scaffold **(C, D)**. **(E)** XRD patterns, **(F)** FTIR spectra, and **(G)** Raman spectra obtained from the COL, GO-COL and CUR-GO-COL scaffolds.

Following the successful loading of curcumin onto graphene oxide and its integration with collagen, a cross-linking treatment was performed to stabilize the composites. The plain collagen scaffold exhibited a white coloration (Figure 2A inset), and the SEM images of pure collagen revealed a network structure, as shown in Figures 2A, B. In contrast, the incorporation of CUR-GO resulted in the CUR-GO-COL composite scaffold displaying a brownish-yellow coloration (Figure 2C inset), with the surface of the composite exhibiting a pronounced roughening effect, as demonstrated in Figures 2C, D. Upon further examination, we observed that the 0.125% (w/v) group (5CUR-GO-COL) exhibited structural discontinuity, while the 0.15% (w/v) group (6CUR-GO-COL) displayed a loose and incomplete formation (Supplementary Figure S7). Consequently, due to the unsuccessful molding of these two groups, we excluded them from subsequent. XRD patterns of COL, GO-COL, and CUR-GO-COL each revealed a broad diffraction peak at a 2θ value of 20°, indicative of the amorphous phase of collagen. The GO-COL and CUR-GO-COL composite scaffolds exhibited a distinctive peak at 10.2°, characteristic of GO. Additionally, the CUR-GO-COL composite scaffold displays a characteristic peak at 17.25°, corresponding to curcumin (Figure 2G). Meanwhile, FTIR spectroscopy confirmed the formation of amide bonds (black line) in the CUR-GO-COL composite scaffold. Raman spectroscopy was utilized to determine the composition of each composite scaffold (Figure 2H). The Raman spectrum of pure COL revealed an absence of distinct peaks, with the curve demonstrating a gradual increase. Conversely, the Raman spectra of GO-COL and CUR-GO-COL exhibited two distinct peaks at 1,348 cm^−1^ and 1,590 cm^−1^, corresponding to the D and G bands of GO, respectively (Figure 2I). The findings of these tests substantiate the presence of graphene oxide within the composite scaffold. Furthermore, it can be observed that the D band in the CUR-GO-CO is more intense compared to the G band and the presence of small peaks were attributed to the loaded curcumin.

### 3.2 Property analysis

**FIGURE 3:**
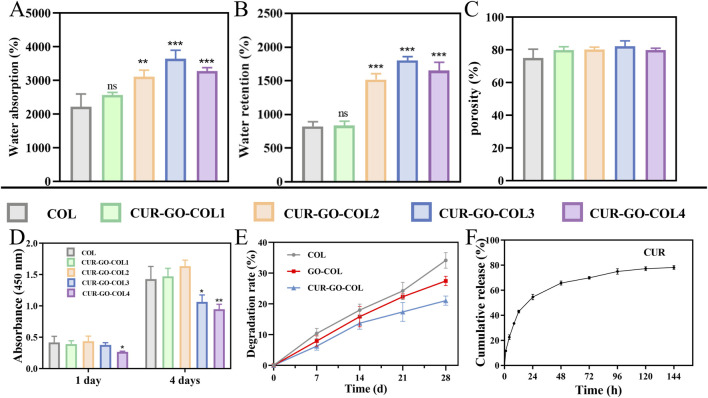
Characterization of the CUR-GO-COL composite scaffold. **(A)** Water absorption, **(B)** water retention and **(C)** porosity obtained from COL, CUR-GO-COL1, CUR-GO-COL2, CUR-GO-COL3 and CUR-GO-COL4 scaffolds. (n = 3; *, *P* < 0.05; **, *P* < 0.01; ***P < 0.001 compared with the COL group). **(D)** CCK-8 assay of cell viability on the COL and CUR-GO-COL scaffolds. (n = 4; *, *P* < 0.05; **, *P* < 0.01; ***P < 0.001 compared with the COL group). **(E)** The degradation performance of the COL, GO-COL and CUR-GO-COL scaffolds. **(F)** Curcumin release from the CUR-GO-COL scaffolds.

In this work, the water absorption of as-prepared scaffolds were analyzed. The natural collagen scaffold demonstrated a water absorption of 2,219.97% ± 379.122%. However, for the CUR-GO-COL composite scaffolds, a trend of initially increasing and then decreasing on water absorption was observed as the increase of CUR-GO concentration. The respective water absorption rates were 2,566.993% ± 77.356%, 3,115.333% ± 189.547%, 3,647.243% ± 250.879% and 3,275.857 ± 104.132 (Figure 3A). Similarly, water retention rates followed the same trend in response to the concentration of collagen. The water retention rate of the collagen scaffold was 823.126% ± 69.519%. In comparison, the water retention rates of the CUR-GO-COL1, CUR-GO-COL2, CUR-GO-COL3 and CUR-GO-COL4 composite scaffolds were found to be 836.806% ± 64.182%, 1,519.967% ± 85.148%, 1801.11% ± 59.180% and 1,651.547% ± 125.153%, respectively (Figure 3B). The porosity measurements indicated that all CUR-GO-COL composite scaffolds with varying concentrations possessed high porosity with values of 79.84586% ± 2.1237%, 80.211% ± 1.537%, 82.170% ± 3.393%, and 79.867% ± 1.155%, separately. No statistically significant difference was observed in porosity when compared to the natural collagen scaffold (75.131% ± 5.345%) (Figure 3C).

### 3.3 Cytotoxicity of the composite scaffolds

The cytotoxicity of the as-prepared CUR-GO-COL composite scaffolds was evaluated using the CCK-8 test. The viability of BMSCs on composite scaffolds with various concentrations of CUR-GO was analyzed on 1 day and 4 days, separately (Figure 3D). The results indicated that at a CUR-GO concentration of 0.75% w/v or higher, a notable reduction in cell viability was observed. The density of the living cells in these groups was significantly lower than that in the other groups. In contrast, when the CUR-GO content was 0.5% (w/v) or lower, the density of living cells was comparable to that observed in the pure COL scaffold group, and the cells proliferated well, indicating that the CUR-GO-COL composite scaffolds within this concentration range did not exhibit significant cytotoxicity to BMSCs. However, the physicochemical properties of biomaterials may regulate the microenvironment of defects and accelerate their healing. Hence, the CUR-GO-COL 2 with better water absorption and retention rate was selected for further study.

### 3.4 *In vitro* degradation property analysis

The degradation rates of various scaffolds were evaluated by weight loss after immersion in PBS for a specified period. As shown in Figure 3E, the degradation rate of the COL group was faster than that of the GO-COL and CUR-GO-COL groups, with a degradation rate of 34.14% ± 2.54% after 28 days. The final degradation rates of the GO-COL and CUR-GO-COL groups were significantly lower than that of the COL group, at approximately 27.43% ± 1.53% and 21.05% ± 1.49%, respectively. These results indicate that the GO-COL and CUR-GO-COL groups exhibit superior stability. Additionally, the degradation rate of the COL group continued to increase over the 28 days, whereas the degradation rates of the GO-COL and CUR-GO-COL groups increased during the first 14 days and then slowed down, demonstrating a more sustained degradation profile.

### 3.5 *In vitro* drug release property analysis

In this research, curcumin served as the main antimicrobial component within the composite scaffolds. The release kinetics of curcumin from these composite scaffolds were quantified using UV-visible spectroscopy and the results are recorded in Figure 3F. Within the first 24 h, a rapid release rate of curcumin from the composite scaffolds was detected with a value of approximately 54.637% ± 2.139%. Beyond 24 h, the rate of curcumin release slowed down. More importantly, the maximum release amount of curcumin from the composite scaffold reached its peak at 78.849% ± 2.195% at 144 h.

### 3.6 *In vitro* cytocompatibility of the CUR-GO-COL scaffold

**FIGURE 4:**
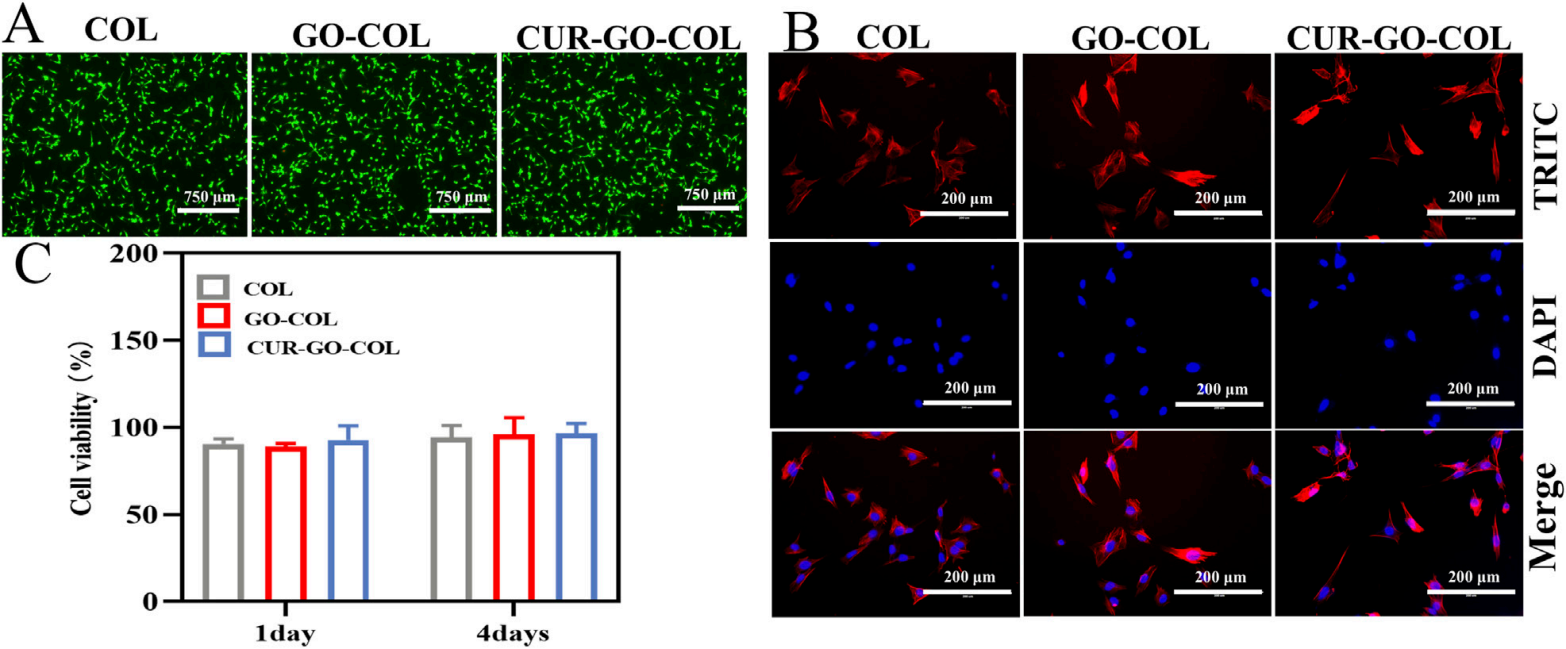
Growth ability of BMSCs cells cultured *in vitro*. **(A)** Live/dead staining images of BMSCs cultured with the COL, GO-COL and CUR-GO-COL scaffolds. **(B)** Immunofluorescence images of BMSCs morphology on the COL, GO-COL and CUR-GO-COL scaffolds BMSCs nuclei stained with DAPI (blue) and the cytoskeleton stained with FITC (red). **(C)** CCK-8 assay of cell viability on the COL, GO-COL and CUR-GO-COL scaffolds at 1 and 4 days (n = 4).

To verify the hypothesis that the bioscaffold materials can provide a suitable microenvironment for supporting bone tissue repair, the cytocompatibility of the CUR-GO-COL composite scaffolds were assessed by conducting the live-dead cell staining experiments. The results, as shown in Figure 4A, indicated that live cells appeared in green while dead cells were in red colour. One day after the inoculation of BMSCs, a large amount of living cells (green) and only a few dead cells (red) were observed across all tested groups. Moreover, the cells were homogeneously distributed and displayed a polygonal morphology, indicating no significant difference in cytocompatibility among the groups.

Here, the cell adhesion morphology was further examined using fluorescence microscopy after 1 day of incubation on each group of composite materials Figure 4B. The cytoskeleton was stained in red by FITC and the nucleus was colored blue with DAPI. Interestingly, in all groups, BMSCs adhered well to the surfaces of the materials, exhibiting even distribution and the formation of cellular bridges. It suggests that the biocompatibility and three-dimensional porous structure of these groups were beneficial for enhancing cell interaction and adhesion. Notably, the addition of drugs enhanced the dispersal area of cells on the surface of composite material, and the cells spread almost completely, promoting a more intense cell interaction. Furthermore, cell flattening can be found, indicative of strong adhesive interactions between the cells and the material surfaces. Further demonstrated that the prepared CUR-GO-COL composite scaffolds would provide a more friendly microenvironment for the growth and differentiation of BMSCs cells.

The effect of different material groups on cell proliferation was further explored using a CCK-8 assay. The cell proliferation of bioscaffold material in each group is shown in the Figure 4C, On the first day of inoculation, the cell proliferation rates of the three composite materials groups did not significantly differences, confirming that the selection of CUR-GO had no inhibitory effect on the growth of BMSCs. By the fourth day of inoculation, more than 88.96% of the cells remained alive, a similar result was observed from the pure COL group. This observation was in the good agreement with the results from the live-dead cell staining. It further suggests that the addition of curcumin and GO within the collagen matrix is not toxic to the cells and present good biocompatibility.

### 3.7 Cell osteogenic differentiation property

**FIGURE 5:**
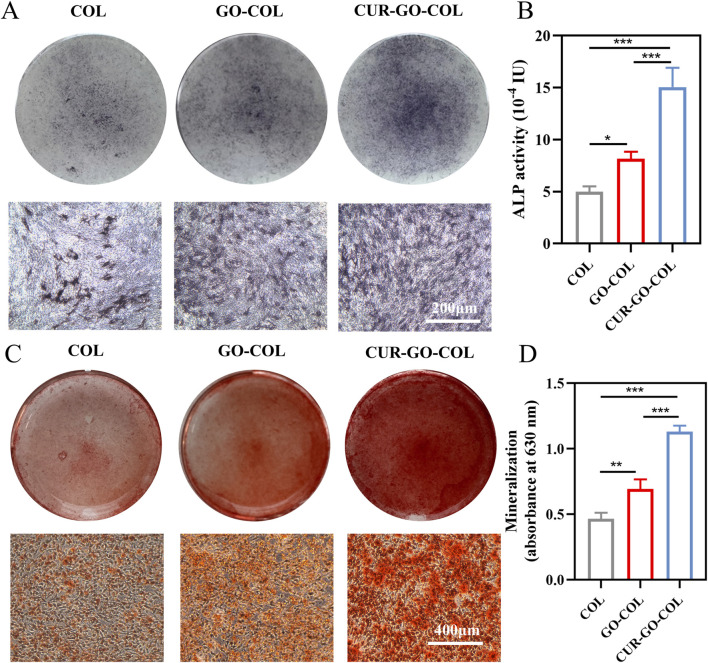
Osteogenic differentiation ability of BMSCs co-cultured with three groups of scaffolds. **(A)** ALP staining. **(B)** ALP activity analysis. **(C)** ARS staining. **(D)** Semi-quantitative analysis. (n = 3; *, *P* < 0.05; **, *P* < 0.01; ***, *P* < 0.001).

To assess the osteogenic differentiation ability of the prepared collagen composite scaffold, the ALP expression level and viability of BMSCs cultured on the composite scaffold, were measured. This is because the expression of ALP representing the early stage of osteogenic differentiation of stem cells. ALP activity of collagen scaffolds in different groups was evaluated according to the color intensity of cells in these groups. The results showed stained cells exhibiting varying shades of purple. Where, CUR-GO-COL group exhibited the darkest ALP staining, followed by the GO-COL group with the COL group showing the least intense staining (Figure 5A). Consistent with the ALP staining results, protein semi-quantitative analysis revealed that the CUR-GO-COL group possessed a higher ALP activity compared to the other two groups, with a statistically significant increase in the GO-COL group relative to the COL group. It indicated that CUR-GO-COL group had good early-stage osteogenic ability and enhanced osteogenic effect with the successful loading of curcumin on GO (Figure 5B).

To further investigate the mineralization of composite scaffold, the expression of calcium nodules in extracellular matrix was detected by alizarin red staining (ARS). Orange mineralized nodules were observed on the surfaces of all cells. While the COL group displayed that the calcium nodules were sparsely distributed. The nodules in the CUR-GO-COL group and GO-COL groups exhibited more numerous and larger, and calcium nodule clusters appeared in the CUR-GO-COL group (Figure 5C). A semiquantitative analysis of the ARS results with 10% cetylpyridinium chloride demonstrated that the CUR-GO-COL and GO-COL groups exhibited significantly increased OD compared to the COL group, where CUR-GO-COL group was followed by GO-COL group (Figure 5D). The enhanced mineralization of the above extracellular matrix suggested that the prepared CUR-GO-COL composite scaffolds can be considered as potential candidates for promoting the expression of late-stage osteogenic markers.

### 3.8 Antibacterial properties of composite scaffold

**FIGURE 6:**
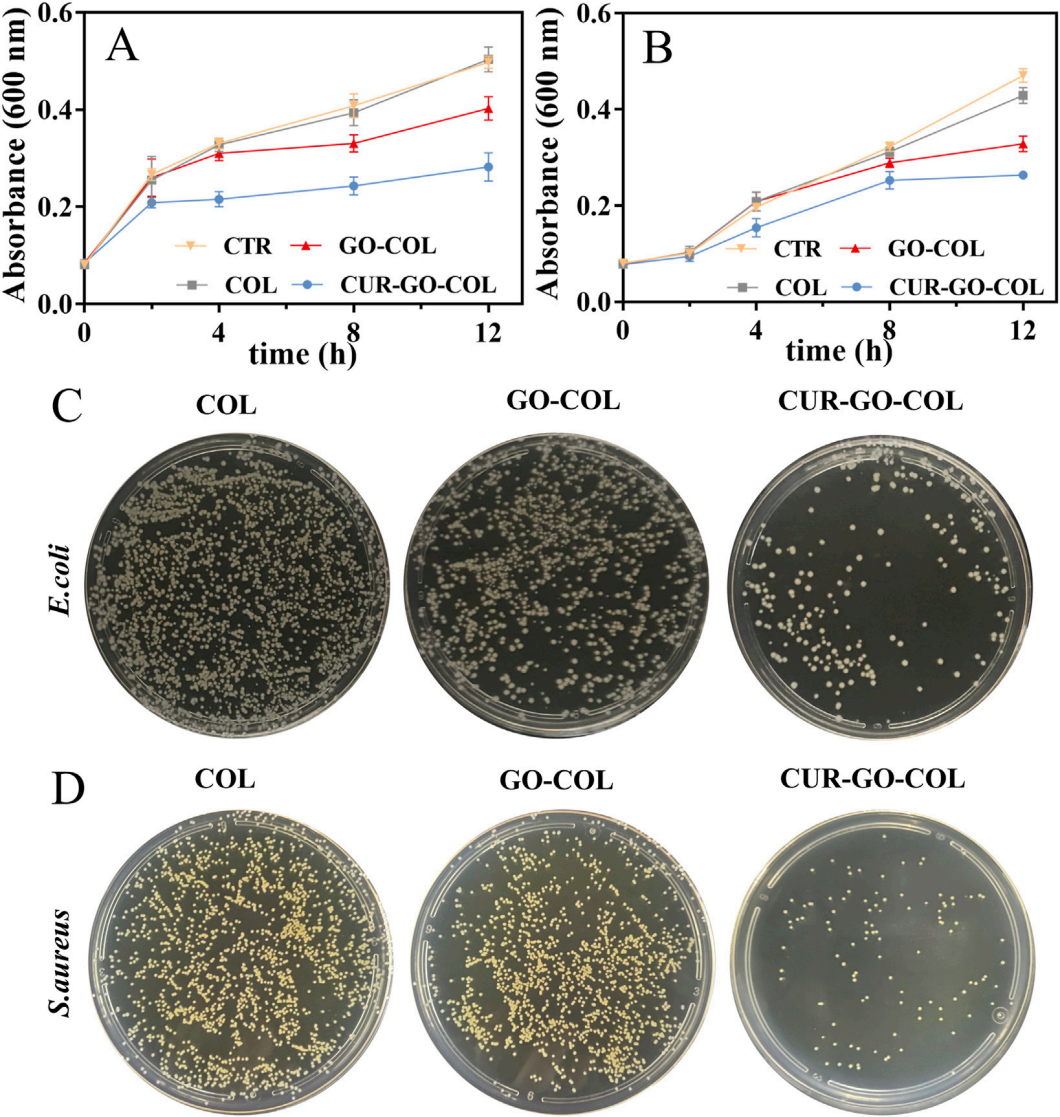
Effect of three groups of scaffolds on bacterial growth. Growth curves **(A)** of **(E)** *coli* and **(B)** *S.aureus*. (n = 3) Plate diffusion results for **(C)** *E. coli and* **(D)** *S.aureus.*

The antibacterial properties of the three scaffold groups were conducted by co-culturing with *S.aureus* and *E.coli*, respectively. The antibacterial activity of the materials was determined by measuring the absorbance value and then monitoring the bacterial growth. As shown in Figures 6A, B, the absorbances (ODs) of bacteria in the COL group were similar to those in the blank group at corresponding time points, proving that the pure COL scaffold did not have obvious antibacterial. In the GO-COL group, the absorbance of bacterial suspension decreased lightly, but the downward trend only became clearly evident after 8 h of co-culture with the bacteria, there was a statistical difference from COL group. The results revealed that the combination with GO alone, exhibited limited antibacterial activity. However, after loading CUR-GO on COL, scaffolds demonstrated a substantial decrease in bacterial absorbance, particularly notable after 12 h of incubation, which was significantly different from the COL group and GO-COL group. This finding concludes that the CUR-GO-COL composite scaffolds could effectively inhibit the growth of the two tested bacteria, showing the excellent antibacterial properties.

To visualize the antibacterial properties of materials in different groups, two strains of bacteria were co-cultured with the material of each group for 24 h. Subsequently, a small amount of bacterial suspension was coated on an agar culture plate, and the antibacterial activity of each group was observed according to the colony growth. As shown in Figures 6C, D, massive bacterial colonies that almost covered overall plates were observed in the COL groups, further proving the lack of antibacterial activity in the pure COL scaffold. After combination of COL with GO, a slight decrease in the colony counts was observed compared to the pure COL group. It indicated that the addition of GO has a slight effect on the bacterial growth inhibition. However, the COL loaded with CUR-GO showed obvious bacterial growth inhibition, proving that the antibacterial properties of the composite material were mainly attributed to the loaded CUR.

## 4 Discussion

The use of natural polymer materials to fill bone defects and promote osteoblast differentiation is a relatively common strategy, but it is also a major challenge (Zhao et al., 2021). While the design and production of collagen scaffolds can fulfil the biological functions of the material, their inadequate mechanical properties and structural instability limit their practical application (He et al., 2024). Curcumin possesses a range of biological activities, including anti-inflammatory, antimicrobial and antioxidant properties, as well as osteogenic capabilities. In order to enhance the functional and mechanical properties of collagen, the curcumin was firstly loaded onto the graphene oxide surface and then the composite was introduced into COL. This combination aims to create a synergistic effect where the loading capacity of GO complements the bioactive functions of curcumin. During the development of the CUR-GO-COL composite scaffolds, the optimal CUR-GO concentration ratio was explored. The CUR-GO-COL composite scaffolds possess biological functions conducive to promoting cellular osteogenic differentiation and antimicrobial ability, according to the investigation into their slow drug release properties, cytocompatibility, osteogenic differentiation ability and antibacterial properties.

The experimental findings indicate that the variation in the CUR-GO concentrations significantly affects the structural integrity, physicochemical properties and bio-compatibility of the CUR-GO-COL composites scaffolds. The stable structure integrity of the composite scaffold edges was observed at specific CUR-GO concentrations. When the CUR-GO concentration reached 0.125% (w/v), a noticeable discontinuity was found at the edges of the composite scaffold. Furthermore, at a slight higher con-centration of 0.15% (w/v), the scaffold presents a loose and unformed structure. This may be due to the excessive addition of CUR-GO in these two groups. Typically, effective crosslinking in composites scaffolds depends on a well-matched ratio of amino groups from collagen and carboxyl groups from GO. Hence, the excessive addition of CUR-GO leads to insufficient crosslinking and ultimately induce the integrity failure of composite scaffold.

The capacity of biomaterials to absorb and retain water plays a crucial role in the absorption of tissue exudates and the transport of nutrients (Dickinson and Gerecht, 2016; Zhang et al., 2016). The water absorption and retention capacities of the composite scaffolds exhibited an initial declining and then ascending trend with the escalation of CUR-GO concentration. The phenomenon can be attributed to the presence of hydrophilic functional groups in graphene oxide, such as carboxyl and hydroxyl groups. These functional groups, due to their geometry, exert a constraining effect on the polymer, leading to an enhanced capacity for water absorption and retention (Kang et al., 2022). However, the CUR-GO-COL4 composite scaffold exhibited reduced water absorption and retention compared with those of the CUR-GO-COL3 composite scaffold. This reduction is likely due to the in-creased curcumin content, which decreases the hydrophilicity of the composite material (Chen et al., 2012). Furthermore, the composite scaffold must possess adequate porosity for facilitating oxygen and nutrient exchange as well as exudate absorption, thereby preventing infection. The ethanol liquid displacement method was employed to measure the porosity of the material, and no statistical difference in porosity was shown among the various composite scaffolds, suggesting that the incorporation of graphene oxide and curcumin had no adverse effect on the pore structure of the collagen scaffold. The highly porous and interconnected structure of the CUR-GO-COL composite scaffold reveals the potential for biomedical applications (Baniasadi et al., 2020).

When the GO-CUR concentration is less than 0.125% (w/v), the morphology of the composite scaffold remains intact and exhibits good hydrophilicity. Although most studies reported that *in vivo* GO would be taken up and degraded by macrophages and finally excreted out of the body through kidneys, as-prepared CUR-GO-COL composite scaffolds need to pass biosafety tests. Therefore, this study first investigated the activity and proliferation of BMSCs on the CUR-GO-COL composite scaffolds. The proliferation ability of BMSCs showed a downward trend at CUR-GO concentrations exceeding 0.075% (w/v), equivalent to the GO content of 0.75 mg/mL. However, Wang et al. found that cell proliferation would be accelerated at a GO concentration of 2 mg/mL; while proliferation of BMSCs was inhibited at a higher concentration (Madeo et al., 2022). This discrepancy can potentially be attributed to the different concentrations of CUR loaded onto GO. With the increase of CUR concentration, it affects not only the cell count but also the cell morphology, and when superimposed CUR to a certain concentration, it is not conducive to the proliferation and differentiation of cells (Sumayya and Kurup, 2017). Combined with physicochemical properties analysis, the hydrophilic properties of biomaterials can modulate the microenvironment of the defect area, thus accelerating the healing process of the defect. Therefore, CUR-GO-COL2 composite scaffold with better hydrophilic properties, emerges as an optimal choice for subsequent *in vivo* and *in vitro* studies. At the same time, the GO-COL scaffold with a GO content of 0.05% (w/v) was selected as the comparison group.

An ideal biomaterial scaffold for implantation should offer ample growth space and support for cells, with degradation matching tissue regeneration. Fast degradation may lead to insufficient support, affecting bone repair stability, while slow degradation may hinder tissue formation, delaying healing (Burg et al., 2000). Consequently, degradation rate is a key factor in bone tissue engineering, impacting repair effectiveness and tissue function restoration. In this study, the COL group exhibited the fastest degradation rate, consistent with previous findings suggesting that the ionic environment of PBS and prolonged immersion likely promote collagen swelling and degradation, thus accelerating mass loss (Toledano et al., 2020). In contrast, the degradation rates of the GO-COL and CUR-GO-COL groups were significantly lower than that of the COL group, likely due to the introduction of GO. The large specific surface area of GO forms a physical barrier, preventing direct contact between water molecules and collagen, thereby slowing the degradation process (Nowroozi et al., 2021). Moreover, strong hydrogen bonding between GO and collagen, as reported in the literature, contributes to structural stability and reduced degradation rates (Kolanthai et al., 2018). This stable structure combined with a corresponding degradation rate facilitates the controlled release of curcumin, thereby enhancing its stability and improving its bioavailability (Radić et al., 2021).

It is noteworthy to mention that the cell proliferation rate among COL, GO-COL and CUR-GO-COL groups showed no significant difference after 1 day and 4 days. This indicates that the addition of CUR-GO does not inhibit cell proliferation, instead, it may enhance both cell proliferation and adhesion capacity. These findings may be attributed to the following reasons: 1) The porous structure of collagen promotes the exchange of oxygen and nutrition between cells growing in the scaffold materials, providing a good microenvironment for cell proliferation (Wang et al., 2018). In addition, the proper pore size and rough surface are more beneficial to cell adhesion and migration (Katebifar et al., 2022). These characteristics ultimately promote attachment, migration and growth of cells (Ruan et al., 2016). 2) GO’s rich oxygen-containing functional groups provide numerous binding sites for serum proteins, significantly improving cell adhesion (Bugli et al., 2018). 3) The slow release of CUR from the CUR-GO-COL composites further enhances the ability of cell proliferation and dispersion. Previous studies also have reported this phenomenon, that the cell proliferation and dispersion increased with the CUR content (Llorente-Pelayo et al., 2020).

ALP is widely recognized as the early markers of differentiation and maturation of BMSCs. The semiquantitative analysis of ALP can reflect the differentiation level of stem cells (Bakkalci et al., 2021). Alizarin red can chelate with the calcium ions produced by BMSCs to form an orange-red alizarin red-calcium complex deposition, and hence it serves as a visual indicator of stem cell mineralization, highlighting the formation of calcium nodules (Ruan et al., 2016). In consistence with the findings from previous studies, GO promotes the osteogenic differentiation of BMSCs. Notably, the GO-COL group presented a higher content of calcium nodules than the COL group. The beneficial effect of GO on osteogenic differentiation may be attributed to its special physicochemical structure which aids in accumulation of osteogenic factors such as cyanate, OPN, and BMP, and promotes the deposition of calcium salts (Son et al., 2018; Zhou et al., 2018). This study further found that the CUR-GO-COL group exhibited a superior osteogenic ability compared to the GO-COL group by loading curcumin. It is reported that curcumin can stimulate the expression of Runx2, ALP, osteocalcin (OCN), and other genes to induce osteogenic differentiation of cells. In addition, curcumin improves ALP activity and mineralization (Li et al., 2020). Li et al. (Shi et al., 2021) found that curcumin induced osteoblastic differentiation of MC3E3-E1 cells after 21 days, the degree of mineralization of extracellular matrix was higher than that of the control group, and the negative effect of oxidative stress on osteogenesis could be reversed. In addition, it is reported that curcumin promotes the osteogenic differentiation of hPDLSCs through EGR1 (Deininger et al., 2022). Therefore, according to the above analysis, the notable outcome observed in the CUR-GO-COL group in this study may be attributed to the synergistic effects of special surface structure of GO and the curcumin, which provide a suitable microenvironment for enhancing the osteogenic differentiation of BMSCs.

To design an ideal bioscaffold material for bone repair, in addition to possessing the good biocompatibility and osteosynthesis induction ability, it should also exhibit a certain inhibition capability on bacterial colonization. These properties are essential for effectively preventing bacterial infection and reducing the use for antibiotics, thus improving the success rate of material implantation. GO and CUR are known for the satisfactory antibacterial properties, but the mechanisms on their antibacterial activity within collagen composites are still not clear. Most infections of the implant are mainly caused by *S. aureus* and *E. coli*, in this study, the antibacterial activities of various bioscaffold materials were evaluated by co-culture with these two bacteria, aiming to investigate the antibacterial mechanism and activity of the materials. The absorbance value (OD value) of the bacterial fluid was measured to represent the bacteria number. The trend of OD value in the COL group was similar to that of the blank control group, indicating that pure collagen did not possess antibacterial properties (Khan et al., 2021). Therefore, the COL group was used as the control in the subsequent experiments. When GO was incorporated, the material exhibited some antibacterial activity, but the outcome was not significantly impactful. According to previous analyses, the antibacterial mechanism of GO was possibly realized by the mechanical damage to bacterial membrane through the sharp edge of GO, which destroys the structural integrity of bacteria, and leads to the bacteria death (Hu et al., 2021; Zamri et al., 2021). However, this mechanism is mainly observed when GO is in suspension state. When GO was homogeneously distributed in polymer matrix, the GO interface control and dispersion are altered, reducing its capacity for the mechanical damage and hence the antibacterial properties (Khan et al., 2020). The antibacterial properties of GO-COL were not obvious in the early phase. However, after 8 h of co-culture, its antibacterial properties were gradually enhanced with slow release of GO from COL. During loading of CUR-GO, a decreased absorbance of bacterial suspension in the early phase is likely due to the rapid release of CUR, thereby resisting the infection by two strains of bacteria. A recent study has shown that curcumin can change the structure of mature multi-species biofilms, significantly decreasing the metabolic activity (Shahzad et al., 2015). It is speculated that CUR would prevent the biofilm formation by influencing bacterial quorum sensing (QS) system, thus preventing the replication of the bacteria (Zheng D. T. et al., 2020). The synergistic effect of CUR and GO enhances antibacterial activity of the collagen composites.

While our study has demonstrated the efficacy of our material within certain parameters, we recognize that a deeper understanding of the mechanisms of material action is necessary for more thorough analysis. However, we acknowledge that further elucidation requires advanced technologies beyond our current resource capacity. Currently, we plan to transplanted the composite material into experimental animals and utilize imaging techniques to monitor the positioning and migration of the material at the bone defect site. Simultaneously, we will assess its degradation rate and biocompatibility through histological analysis and biochemical testing. Additionally, we will observe the impact of the composite material on the bone healing process, including the rate and quality of new bone formation. This series of studies will provide scientific evidence for the application prospects of the composite material in the field of bone defect repair, evaluating its potential and effectiveness as a bone substitute.

## 5 Conclusion

In this study, a novel multifunctional CUR-GO-COL composite scaffold was synthesized for bone tissue repair. Among them, the collagen component COL can improve the hydrophilicity of the composite scaffold; GO can delay the release of curcumin and improve its bioavailability. In addition, the cytotoxicity, osteogenic differentiation and antibacterial properties of the collagen composite scaffold were evaluated. The composite scaffold attained the optimal physico-chemical performance and biological safety with a CUR-GO concentration of 0.05% (w/v). Therefore, the resulting porous CUR-GO-COL composite scaffold with structure stability supports prolific cell growth without cytotoxicity, effectively inhibits bacterial colonization, and reduces the need for systemic antibiotics, making it a promising candidate for bone grafts.

## Data Availability

The original contributions presented in the study are included in the article/Supplementary Material, further inquiries can be directed to the corresponding author.
