# Supplementary material for: Biological and structural properties of curcumin-loaded graphene oxide incorporated collagen as composite scaffold for bone regeneration
Source: Front Bioeng Biotechnol. 2024 Nov 20;12:1505102. doi: 10.3389/fbioe.2024.1505102 (PMC11614606; doi:10.3389/fbioe.2024.1505102)
Supplement: Supplementary file 1 [file DataSheet1.pdf]

Supplementary Material

1 Supplementary methods

Selection of curcumin determination wavelength and standard curve preparation

Firstly, a curcumin standard solution was prepared. 8 mg of curcumin was accurately weighed and fully dissolved in 10 ml of anhydrous ethanol. Subsequently, varying volumes of the solution were transferred into 10 ml volumetric flasks and diluted to the mark with anhydrous ethanol. Using anhydrous ethanol as a blank control, the curcumin solutions at different concentrations were placed in cuvettes and subjected to UV scanning in the wavelength range of 300-500 nm using a UV-Vis spectrophotometer. This generated UV absorption spectra of each curcumin solution, thereby determining the absorption wavelength of curcumin. At this wavelength, the absorbance of different curcumin concentrations was measured, and a standard curve was plotted with curcumin concentration as the x-axis and absorbance as the y-axis.

2 Supplementary Figures

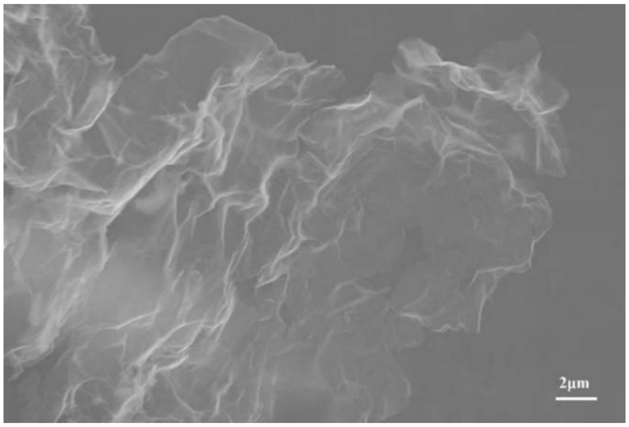

Figure S1. SEM image of a GO sheet.

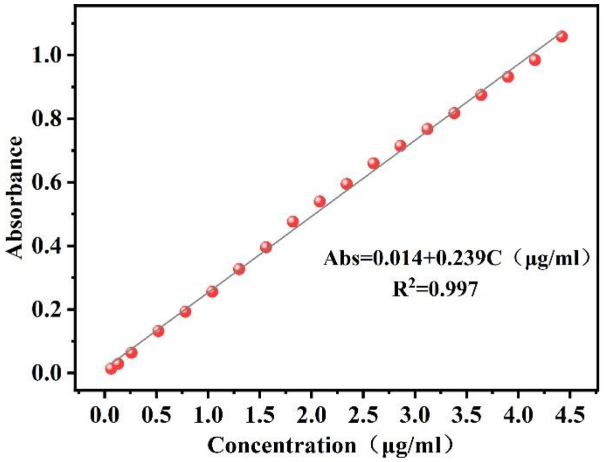

Figure S2. Standard curve of curcumin solutions.

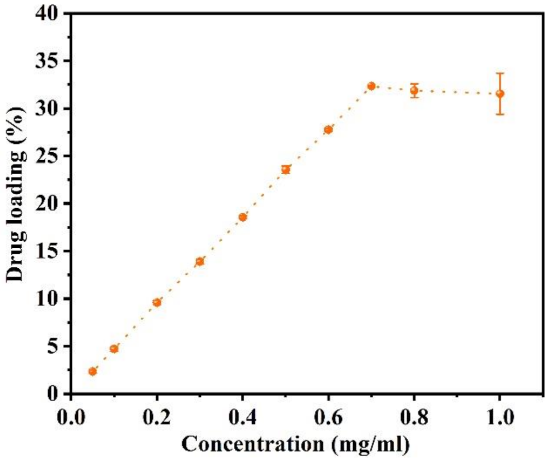

Figure S3. Loading rate of curcumin solutions.

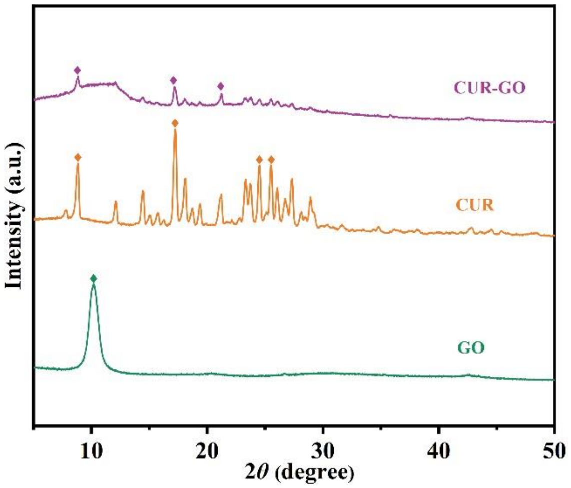

Figure S4. XRD patterns obtained from GO, curcumin and CUR-GO samples.

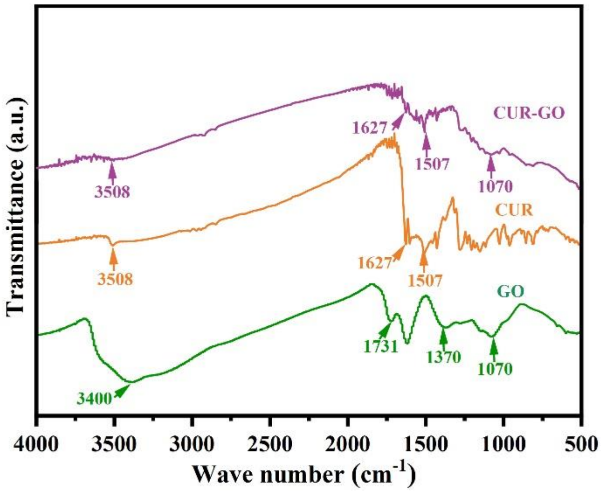

Figure S5. FTIR spectra obtained from GO, curcumin and CUR-GO samples.

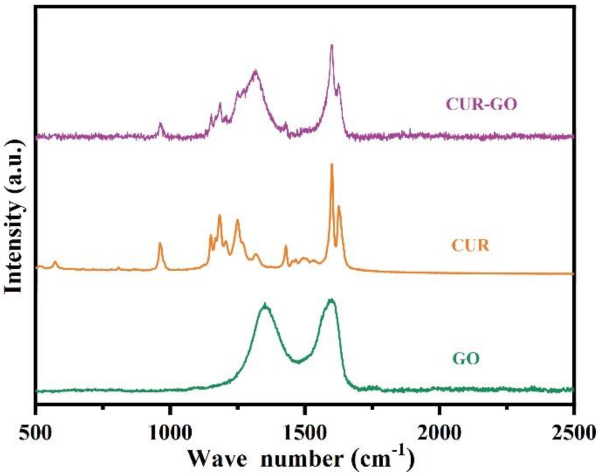

Figure S6. Raman spectra obtained from GO, curcumin and CUR-GO samples.

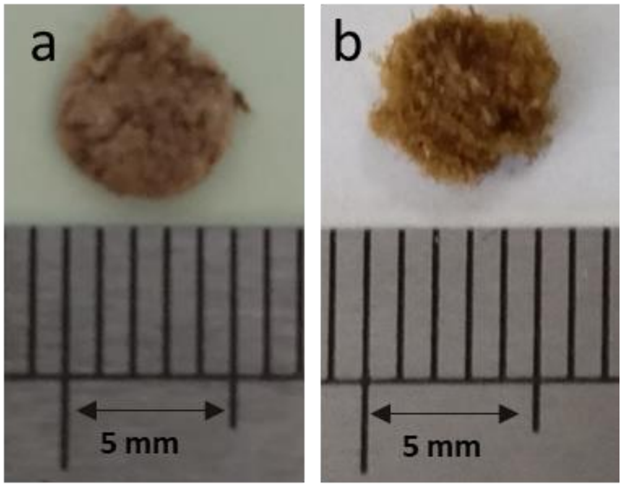

Figure S7. Optical images obtained from as-prepared CUR-GO-COL membranes. (a)0.125%(W/V) (5 CUR-GO-COL), (b) 0.15%(W/V) (6 CUR-GO-COL)
